# Rosetting Plasmodium falciparum-Infected Erythrocytes Bind to Human Brain Microvascular Endothelial Cells *In Vitro*, Demonstrating a Dual Adhesion Phenotype Mediated by Distinct P. falciparum Erythrocyte Membrane Protein 1 Domains

**DOI:** 10.1128/IAI.01233-13

**Published:** 2014-03

**Authors:** Yvonne Adams, Pongsak Kuhnrae, Matthew K. Higgins, Ashfaq Ghumra, J. Alexandra Rowe

**Affiliations:** a Centre for Immunity, Infection and Evolution, Institute of Infection and Immunity, School of Biological Sciences, University of Edinburgh, Edinburgh, United Kingdom; b Microbiology Department, King Mongkut's University of Technology Thonburi, Bangkok, Thailand; c Department of Biochemistry, University of Oxford, United Kingdom

## Abstract

Adhesion interactions between Plasmodium falciparum-infected erythrocytes (IE) and human cells underlie the pathology of severe malaria. IE cytoadhere to microvascular endothelium or form rosettes with uninfected erythrocytes to survive *in vivo* by sequestering IE in the microvasculature and avoiding splenic clearance mechanisms. Both rosetting and cytoadherence are mediated by the parasite-derived IE surface protein family Plasmodium falciparum erythrocyte membrane protein 1 (PfEMP1). Rosetting and cytoadherence have been widely studied as separate entities; however, the ability of rosetting P. falciparum strains to cytoadhere has received little attention. Here, we show that IE of the IT/R29 strain expressing a rosette-mediating PfEMP1 variant (IT4var09) cytoadhere *in vitro* to a human brain microvascular endothelial cell line (HBEC-5i). Cytoadherence was inhibited by heparin and by treatment of HBEC-5i with heparinase III, suggesting that the endothelial receptors for IE binding are heparan sulfate proteoglycans. Antibodies to the N-terminal regions of the IT4var09 PfEMP1 variant (NTS-DBL1α and DBL2γ domains) specifically inhibited and reversed cytoadherence down to low concentrations (<10 μg/ml of total IgG). Surface plasmon resonance experiments showed that the NTS-DBLα and DBL2γ domains bind strongly to heparin, with half-maximal binding at a concentration of ∼0.5 μM in both cases. Therefore, cytoadherence of IT/R29 IE is distinct from rosetting, which is primarily mediated by NTS-DBL1α interactions with complement receptor 1. These data show that IT4var09-expressing parasites are capable of dual interactions with both endothelial cells and uninfected erythrocytes via distinct receptor-ligand interactions.

## INTRODUCTION

The malaria parasite Plasmodium falciparum is capable of avoiding clearance by the spleen via means of sequestration, whereby infected erythrocytes (IE) adhere to endothelial cells lining microvascular blood vessels via various host receptors (reviewed in reference 1). The ability to sequester within the host's tissues is mediated by members of the highly variable protein family Plasmodium falciparum erythrocyte membrane protein 1 (PfEMP1), expressed on the surface of IE (2–4). PfEMP1 variants are high-molecular-mass (200 to 400 kDa) proteins containing multiple extracellular domains, characterized as Duffy-binding like (DBL) and cysteine-rich interdomain regions (CIDR) (5). Some of these PfEMP1 domains have been shown to mediate specific adhesive interactions with host receptors, e.g., CIDRα domains bind to CD36 (6) while DBL2β-C2 from some PfEMP1 variants binds to ICAM-1 (7).

Parasite adhesion interactions are not restricted to the endothelium; IE can bind uninfected erythrocytes to form rosettes (8) or platelets to form platelet-mediated clumps (9). Rosetting is an important parasite adhesion phenotype that has been associated with virulence and severe malaria in multiple studies (10–12). However, the behavior and localization of rosetting parasites *in vivo* is poorly understood. Rosettes are not seen in the peripheral bloodstream, suggesting that rosetting parasites are sequestered in the microvasculature. It is unclear whether this is due to direct binding of rosetting IE to endothelial cells or to binding of rosetting parasites to nonrosetting cytoadherent IE (a rosetting IE can bind to both uninfected and infected red cells). Limited literature exists regarding the ability of rosetting parasites to undergo direct adhesion to microvascular endothelial cells. In 2003, Vogt and colleagues suggested that rosetting parasites of strain FCR3S1.2 bound to the glycosaminoglycan heparan sulfate on endothelial cells (13). The parasite ligand for binding was suggested to be the DBLα domain of PfEMP1 variant FCRS1.2var1, which mediated a dual rosetting and endothelial cell binding phenotype (13). However, direct adhesion of rosetting IE to endothelial cells was not demonstrated, and recently a different PfEMP1 variant, IT4var60, has been identified as the rosetting ligand in FCR3S1.2 parasites (14). The FCR3S1.2var1 gene may instead be transcribed by nonrosetting parasites within FCR3S1.2, as parasite populations always contain a mixture of PfEMP1 variants, even when selected for a single adhesion phenotype, due to spontaneous *var* gene switching *in vitro*. Therefore, the significance of Vogt et al.'s findings in terms of rosetting parasites is unclear. No other studies have yet been published on rosetting parasite interactions with microvascular endothelial cells.

Here, a well-characterized P. falciparum rosetting strain, IT/R29 (15), was investigated for its ability to bind to a human brain microvascular endothelial cell line (HBEC-5i) (16). Adhesion to HBEC-5i was demonstrated, the adhesion-blocking effects of antibodies to specific PfEMP1 domains were examined, and the role of heparan sulfate as an endothelial cell receptor was investigated.

## MATERIALS AND METHODS

### Parasite strains.

The rosetting laboratory clone IT/R29 (17), derived from the IT/FCR3 strain, was the main focus of this study. The rosetting characteristics of this parasite, including identification of the IT4var09 PfEMP1 variant as the parasite rosetting ligand (15, 18), identification of complement receptor 1 (CR1) as the major uninfected erythrocyte rosetting receptor (15, 19), and rosette disruption by sulfated glycoconjugate compounds (20, 21), have been described previously. Two nonrosetting parasite strains were used as positive controls for HBEC-5i binding. They were FCR3-CSA, which was selected for binding to chondroitin sulfate A (CSA) (22), a molecule that is well expressed on HBEC-5i (23), and HB3-HBEC, which was selected for adhesion to HBEC-5i and binds to an unknown endothelial receptor (24). A negative-control parasite strain, IT unselected (IT/Uns), that is unable to bind to HBEC-5i, was also used. IT/Uns was derived from clone IT/A4 (17) grown without selection for >20 generations. IT/Uns binds well to CD36 but poorly to other receptors (25), and it is unable to bind HBEC-5i, as these cells lack CD36.

### Parasite culture.

Plasmodium falciparum IE were maintained in culture with O^+^ erythrocytes (Scottish National Blood Transfusion Service) in RPMI 1640 medium (Invitrogen) containing 25 mM sodium bicarbonate, supplemented with 5% pooled human serum (Scottish National Blood Transfusion Service), 2 mM l-glutamine (Invitrogen), 25 mM HEPES (Lonza), 20 mM glucose (Sigma), 25 μg/ml gentamicin sulfate (Lonza), and 0.25% Albumax II (Gibco). The pH was adjusted to 7.2 to 7.4 with the addition of NaOH (Sigma), and flasks were kept at 37°C and gassed with 1% O_2_–3% CO_2_–96% N_2_. Parasite maturity and parasitemia were assessed by daily thin blood smears stained with 10% Giemsa.

### Assessment of RF.

The rosette frequency (RF) of IT/R29 parasites was determined by viewing a wet preparation of 25 μg/ml ethidium bromide-stained culture suspension (10 μl on a clean microscope slide covered by a 22- by 22-mm coverslip) using simultaneous white light and fluorescence to visualize both infected and uninfected erythrocytes (40× objective; Leica DM LB2 microscope). A rosette was defined as an IE that binds two or more uninfected erythrocytes. The RF is the percentage of IE forming rosettes out of 200 IE counted. For all assays, the RF was calculated in triplicate for a total of 600 cells from three independent wet preparations per sample either prior to assay (trophozoite start) or during the previous cycle (ring start), and parasites with an RF of at least 60% were used in all experiments.

### HBEC-5i culture.

HBEC-5i is an immortalized cell line (16) that has recently been used as an *in vitro* model for the host-parasite interactions underlying cerebral malaria and sequestration of IE in the human brain (24–26). HBEC-5i were seeded onto 50 μg/ml fibronectin (Millipore)-treated flasks and cultured in Dulbecco's modified Eagle medium (DMEM)-F12 medium (Sigma) supplemented with 10% heat-inactivated fetal bovine serum (FBS), 2 mM l-glutamine (Invitrogen), 10 μg/ml gentamicin sulfate, and 5 ml endothelial growth supplement (ScienCell) (16). For adhesion assays, cells were seeded onto fibronectin-coated 8-well chamber slides for a minimum of 48 h prior to the assay (24, 25). The fibronectin-coated chamber slides were either purchased (BD Biosciences) or coated manually with 50 μg/ml fibronectin (Millipore) for 10 min at 37°C prior to seeding with HBEC-5i. Parasite and HBEC-5i cultures were checked regularly for mycoplasma contamination (27), and only mycoplasma-negative cultures were used for experiments.

### Adhesion assays. (i) Pigmented trophozoite start.

Pigmented trophozoite IE were resuspended at 1% hematocrit and 5% parasitemia in bicarbonate-free RPMI 1640 (pH 7.2) containing 2 mM l-glutamine and 25 mM HEPES (Lonza) and supplemented with 10% human serum, (Invitrogen), 20 mM glucose (Sigma), and 25 μg/ml gentamicin sulfate (Lonza). The IE (500 μl per well; 1 cm^2^) were added to HBEC-5i cell monolayers (>70% confluence) grown in 8-well chamber slides. Chamber slides were then gassed in a humidified incubation chamber for 2 min with 1% O_2_–3% CO_2_–96% N_2_ and then incubated at 37°C for 1 h with gentle agitation every 15 min. After 1 h, the chambers were removed per the manufacturer's instructions, and washing was carried out by gently immersing the slides upright in a staining jar with 50 ml prewarmed (37°C) washing buffer (RPMI 1640 without bicarbonate, pH 7.2) for 30 min. Adherent IE were fixed in 2% glutaraldehyde in phosphate-buffered saline (PBS) (Sigma) for 1 h before being stained with 5% Giemsa for 15 min. The number of bound IE in 10 fields was counted using a Leica DM 2000 microscope (40× magnification) and expressed as the mean number of bound IE per mm^2^ for at least three independent experiments.

### (ii) Ring-stage start.

IE at ring stage were adjusted to 5% parasitemia and 1% hematocrit in HBEC-5i medium as described above, and 500 μl of parasite suspension was added to each well containing adherent monolayers of HBEC-5i in 8-well chamber slides. The brain endothelial cells and IE were coincubated overnight at 37°C in 5% CO_2_ to allow for optimum growth conditions for the HBEC-5i. Pilot experiments determined that the parasites showed normal morphology and maturity when grown under these conditions. The following day, the chambers were carefully removed and nonadherent IE washed off by inversion into a petri dish (90-mm diameter) containing 10 ml prewarmed wash buffer (RPMI 1640 without bicarbonate, pH 7.2) for 30 min. This was done by gently placing the inverted slides onto Eppendorf tube lids, cut from tubes, to suspend them above the bottom of the petri dish and allow the removal of nonadherent cells by gravity. Manual washing of chamber slides in a staining jar was too vigorous for the adherent IT/R29; therefore, washing by gravity preserved the bound IE for fixation. Cells were then fixed in 2% glutaraldehyde and stained with Giemsa as described above. The number of bound IE was counted (10 fields with 40× objective) using a Leica DM 2000 microscope and expressed as the mean number of bound IE per mm^2^ for at least three independent experiments.

### Human primary endothelial cell line adhesion assays.

Primary human brain microvascular endothelial cells (HBMEC) and human pulmonary microvascular endothelial cells (HPMEC) were purchased from ScienCell and cultured by following the manufacturer's instructions. Briefly, cells were grown in fibronectin-treated flasks at 37°C in 5% CO_2_ using endothelial cell medium (ScienCell) supplemented with 2 mM l-glutamine, 5 ml penicillin-streptomycin (100×), 5 ml endothelial growth supplement (100×; ScienCell), and 5% heat-inactivated fetal bovine serum (Gibco). For adhesion assays, cells were seeded onto fibronectin-coated 8-well chamber slides for a minimum of 72 h prior to the assay, as described above for HBEC-5i. Ring-stage start adhesion assays were carried out as described above, except endothelial cell medium was used rather than DMEM-F12.

### Antibody production.

Rabbit polyclonal antibodies against recombinant proteins representing individual domains of the IT4var09 PfEMP1 variant were generated as previously described (18). Total IgG was purified as described previously (18) and used over a range of concentrations from 1 to 100 μg/ml. It was not possible to express the CIDRγ domain of IT4var09 as a soluble protein (18); therefore, antibodies to this domain were not tested.

### Immunofluorescence assay (IFA) on fixed cells.

A ring-stage adhesion assay was conducted as described above, and after the final gravity wash, slide chambers were removed per the manufacturer's instructions. The slides were fixed in ice-cold acetone-methanol (90:10) and allowed to return to room temperature for 30 min. Primary incubation was with 50 μl/well of IT4var09 anti-NTS-DBL1α and anti-DBL2γ and negative controls (nonimmunized rabbit IgG and antibody to an irrelevant PfEMP1 variant, NTS-DBL1α of TM180var1 [28]). One mg/ml total IgG stock of each antibody was diluted 1:5,000 in PBS–1% BSA to give a final concentration of 0.2 μg/ml, and incubation was for 1 h at room temperature in a humidified box. Cells were washed 3 times with 50 μl PBS/well for 5 min each time, and then the secondary antibody (Alexa Fluor 488 goat-anti-Rabbit IgG; A-11034; Invitrogen) was added at 1:1,000 dilution in PBS–1% BSA containing 1 μg/ml 4,6-diamidino-2-phenylindole (DAPI) to stain parasite nuclei and incubated for 45 min as described above. A final wash was conducted (50 μl per well for 5 min each wash) before mounting with 1.25 mg/ml DABCO (1,4-diazabicyclo[2.2.2]octane) in 50% glycerol–50% PBS (29).

### Adhesion inhibition assays.

For inhibition assays with antibodies, anti-NTS-DBL1α and anti-DBL2γ were tested at 1, 10, and 100 μg/ml, while anti-DBL3ε, anti-DBL4δ, and anti-CIDR2β antibodies were tested at 100 μg/ml only. Immature ring-stage IT/R29 IE (500 μl) at 5% parasitemia and 1% hematocrit (in HBEC-5i media) were added to fibronectin-coated 8-well chamber slides containing >70% confluent HBEC-5i with the appropriate concentration of antibody. The chamber slides were then incubated overnight at 5% CO_2_ and 37°C to allow for optimum growth of the HBEC-5i cells and allow adhesion to occur. The following day, the chambers were carefully removed and nonadherent IE washed off by gravity and then fixed and stained as described above. The level of binding was determined by counting the number of bound IE per mm^2^ as described above for at least three independent experiments.

### Adhesion reversal assays.

To measure the ability of the PfEMP1 antibodies and compounds to reverse adhesion, assays were set up with ring-stage IT/R29 IE (500 μl) at 5% parasitemia and 1% hematocrit (in HBEC-5i media) being added to fibronectin-coated 8-well chamber slides containing >70% confluent HBEC-5i cells. The chamber slides were then incubated overnight at 5% CO_2_ and 37°C to allow adhesion to occur. The following day, anti-NTS-DBL1α and anti-DBL2γ antibodies were tested at 1, 10, and 100 μg/ml, while anti-DBL3ε, anti-DBL4δ, and anti-CIDR2β antibodies were tested at 100 μg/ml only. In addition, a panel of sulfated glycoconjugates (heparin, heparan sulfate, fucoidan, CSA, CSB, hyaluronic acid [all from Sigma], and curdlan sulfate [30]) were also tested at 100 μg/ml. For all adhesion reversal assays, the medium within the 8-well chamber slides was removed with a P1000 pipette, taking care not to disturb the settled cells, and prewarmed HBEC-5i medium containing the appropriate test compound/antibody was added and slides were then gently agitated to mix the cells. Once all compounds were added, the chamber slides were returned to the 5% CO_2_, 37°C incubator for 1 h. After 1 h, the chambers were carefully removed and nonadherent IE washed off by gravity wash as described above. Cells were then fixed in 2% glutaraldehyde and stained with Giemsa as described above. The adherent cells were counted and expressed as the mean number of IE bound per mm^2^ for at least three independent experiments.

### Enzymatic treatment of HBEC-5i.

To remove heparan sulfate from the surface of the HBEC-5i, adhesion assays were performed as described above with ring-stage IT/R29 IE using a protocol similar to that of the adhesion reversal assay described above, with some modifications. Previous assays used fibronectin-coated slides; however, heparinase III treatment caused the cells to lift off; therefore, HBEC-5i cells were seeded onto 0.1% gelatin-coated slides (in PBS for 2 h at 37°C), and the assays were continued as reported above. After the overnight incubation, medium was carefully removed from the wells of the chamber slide and replaced with 500 μl HBEC-5i medium containing either 0.2 IU/ml heparinase III (Sigma), 0.5 IU/ml chondroitinase ABC (Sigma), or no enzyme (control). The 8-well chamber slides were then placed in a humidified box, gassed with 1% O_2_–3% CO_2_–96% N_2_, and incubated at 27°C, the optimum temperature for heparinase III activity, for 2 h. After 2 h, the chambers were carefully removed and nonadherent IE washed off, as described above, by gravity wash. Cells were then fixed in 2% glutaraldehyde and stained with Giemsa as described above. The level of adhesion was determined by counting the number of bound IE per mm^2^ as described above for at least two independent experiments. An additional control for enzymatic treatment was performed by pretreating HB3-HBEC IE (whose adhesion to HBEC-5i is not heparan sulfate dependent [25]) with either heparinase III or chondroitinase ABC to exclude nonspecific enzyme effects.

### SPR analysis of heparin binding.

Proteins were expressed and purified as described previously (18), before dialysis into 20 mM HEPES, pH 7.5, 150 mM NaCl, 0.005% Tween 20. Biotinylated heparin was prepared by dissolving porcine intestinal heparin (Sigma) to 5 mg/ml in 20 mM sodium phosphate, pH 7.4, 150 mM NaCl. One ml of this was incubated with 25 μl of 50 mM biotin-hydrazide (Pierce) and 15 μl of 100 mg/ml 1-ethyl-3-(3-dimethylaminopropyl) for 2 h at 25°C. The biotinylated heparin was then dialyzed overnight into 20 mM sodium phosphate, pH 7.4, 150 mM NaCl.

Measurements were performed on a Biacore 2000 instrument with a constant flow rate of 30 μl/min. Biotinylated heparin (100 μl) was injected over flow cell 2 of a streptavidin-coated SA chip (Biacore) before channels 1 and 2 were equilibrated with 20 mM HEPES, pH 7.5, 150 mM NaCl, 0.005% Tween 20. IT4var09 PfEMP1 recombinant proteins (18) were injected over both flow cells, and the level of specific binding was obtained from a subtraction of the response from channel 2 from that of channel 1. After each injection, both channels were regenerated with a 30-μl injection of 2 M NaCl, restoring the signal to original levels without damaging the surface.

Data were analyzed using the BIAevaluation software to obtain maximum response values for saturated curves. These were plotted against the concentration of protein using Prism 5.01 (GraphPad Software Inc., CA) and fitted to a single-site binding model, allowing determination of the concentration that gave a half-maximal response. Errors are the given values with 95% confidence.

For competition studies, carbohydrates were dissolved in 20 mM HEPES, pH 7.5, 150 mM NaCl, 0.005% Tween 20 to 1 mg/ml and filtered through a 0.2-μm membrane (Sartorius). These stocks were mixed with recombinant IT4var09 DBL2γ domain (18) to a final concentration of 1 μM and a range of carbohydrate concentrations (1 to 50 μg/ml). These were incubated for a minimum of 30 min before Biacore measurements were taken as described above.

### Adhesion inhibition assays with CR1 mouse MAbs.

Mouse monoclonal antibodies (MAbs) J3D3 (Immunotech) and J3B11 (15, 19) and an IgG1 isotype control (Dako) at 1 μg/ml and 0.1 μg/ml were added to fibronectin-coated 8-well chamber slides containing >70% confluent HBEC-5i. Ring-stage IT/R29 IE (500 μl/well) at 5% parasitemia and 1% hematocrit in HBEC-5i media were added, and the chamber slides were incubated overnight at 37°C with 5% CO_2_. The following day, the chambers were carefully removed and nonadherent IE washed off by gravity and then fixed, stained, and counted as described above.

### Graphing and statistical analysis.

GraphPad Prism v5.0 was used for production of graphs and statistical analysis. Mean values for binding with and without antibodies and compounds were compared by one-way analysis of variance (ANOVA) with Tukey's *post hoc* test. *P* values of <0.05 were taken to be statistically significant.

## RESULTS

### IT/R29 rosetting IE bind to HBEC-5i when adhesion assays are started at ring stage.

**FIG 1:**
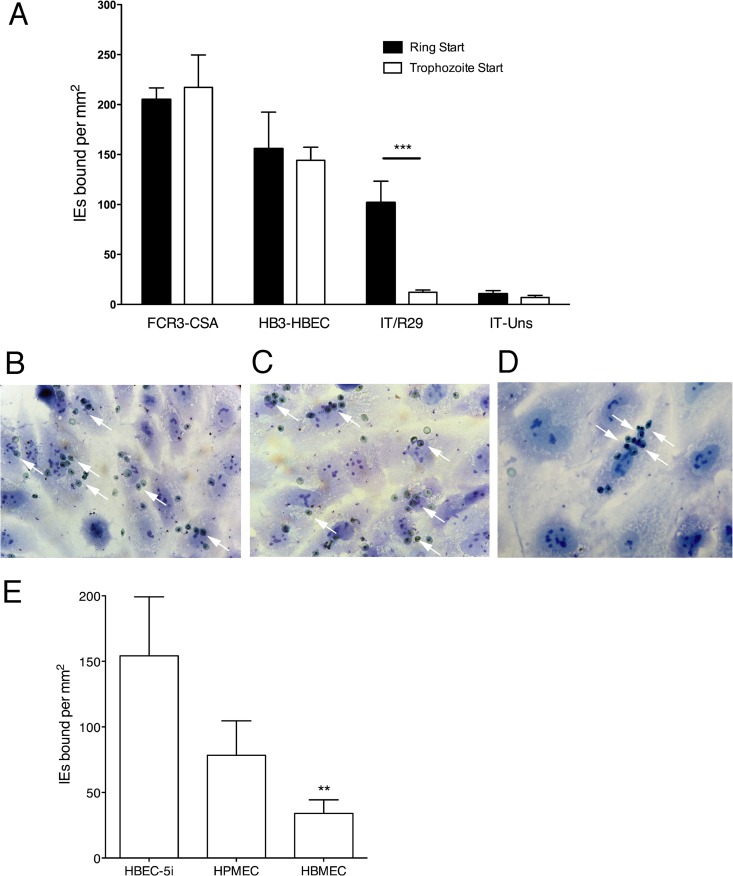
IT/R29 IE bind to HBEC-5i in ring-stage start assays. (A) Comparison of adhesion to HBEC-5i starting with pigmented trophozoite-stage-infected erythrocytes (IE) (white bars) or ring-stage IE (black bars) for parasite strains FCR3-CSA, HB3-HBEC, IT/R29, and IT-Uns. For the trophozoite-stage start assay, P. falciparum mature pigmented trophozoites at 5% parasitemia were incubated with HBEC-5i grown in 8-well chamber slides for 1 h before unbound IE were removed by gravity wash, fixed with 2% gluteraldehyde, and stained with Giemsa. For ring-stage start assay, IE at 5% parasitemia were coincubated overnight with HBEC-5i cells. The following day, unbound IE were removed by gravity wash and fixed and stained as described for the trophozoite-stage start assay. IE adhesion was assessed by microscopic examination of 10 fields (40× objective), and the mean numbers of IE bound and standard errors from four independent experiments are shown. The nonrosetting parasite strains FCR3-CSA, HB3-HBEC, and IT-Uns showed no difference in adhesion between ring-stage and pigmented trophozoite-stage start, whereas the rosetting IT/R29 strain showed significantly higher levels of adhesion after ring stage start (***, *P* < 0.005 by one-way ANOVA with Tukey's *post hoc* test). FCR3-CSA (B) and HB3-HBEC (C) adhesion to HBEC-5i show a diffuse scattering of adherent IE (white arrows). (D) IT/R29 adherent IE occur in clusters (white arrows). (E) Adhesion of IT/R29 IE to primary human pulmonary microvascular endothelial cells (HPMEC) and primary human brain microvascular endothelial cells (HBMEC) in ring-stage start assays as described for panels A and B. The mean numbers of IE bound and standard errors from three independent experiments are shown. Binding to HBMEC was significantly lower than that to HBEC-5i (**, *P* < 0.01 by one-way ANOVA with Tukey's *post hoc* test).

Using standard adhesion assays with mature pigmented trophozoite IE, the positive-control parasite strains FCR3-CSA (mean, 217.2 ± 32.4 [SD] IE per mm^2^; *n* = 4) and HB3-HBEC (144.2 ± 13.1 IE per mm^2^; *n* = 4) both bound well to HBEC-5i cells, as expected (Fig. 1A, white bars). The negative-control parasite strain IT/Uns, which binds primarily to CD36 (25), a molecule that is not expressed on HBEC-5i, showed minimal binding (7.0 ± 2.15 IE per mm^2^; *n* = 4). Under the same conditions, rosetting IT/R29 IE did not bind to HBEC-5i (12.2 ± 2.29 IE per mm^2^; *n* = 4) (Fig. 1A, white bars). This might be because IT/R29, like IT/Uns, is unable to bind to any receptors on HBEC-5i. Alternatively, it is possible that IT/R29 IE are able to bind, but the uninfected erythrocytes in rosettes block access of IE surface molecules to endothelial receptors. To differentiate between these two possibilities, we initially tried using magnetically activated cell sorting (MACS)-purified IT/R29 IE in order to test their adhesion in the absence of uninfected erythrocytes. However, immediately after purification we found that the rosetting IE bound to other IE in the cell suspension to form enormous aggregates of IE that were unable to bind to endothelial cells. Similarly, attempts to disrupt the rosettes prior to adhesion by mechanical means were unsuccessful, because the rosettes reformed immediately.

An alternative approach was to start the assay at ring stage, prior to the expression of surface PfEMP1 and the onset of rosetting (31), by coculturing IE and HBEC-5i. We reasoned that this would allow the IE to come into contact with the HBEC-5i before rosetting begins and allow any potential adhesion interactions with HBEC-5i to be seen. Using the HBEC-adherent positive-control parasites FCR3-CSA and HB3-HBEC, IE cocultured overnight from ring stage bound to HBEC-5i at levels comparable to those measured in the standard pigmented trophozoite start assay (Fig. 1A, black bars) (no significant difference between ring start and trophozoite start in each case; paired *t* test). Similarly, with the negative-control parasite IT/Uns, incubating overnight from ring stage with HBEC-5i did not significantly change the levels of adhesion seen (Fig. 1A, black bars). However, overnight coincubation of IT/R29 IE and HBEC-5i cells allowed adhesion of IT/R29 to be detected and quantified (101.8 ± 21.4 IE per mm^2^; *n* = 4) (Fig. 1, black bars). This was significantly higher than the adhesion observed when the assay was started at pigmented trophozoite stage (*P* = 0.004 by paired *t* test). While the overall level of binding of IT/R29 IE was lower than that of FCR3-CSA and HB3-HBEC, the ability of IT/R29 to bind was consistent and reproducible. The pattern of binding shown by IT/R29 IE differed from that of the other two strains. For FCR3-CSA and HB3-HBEC, the bound IE were evenly dispersed over the lawn of HBEC-5i (Fig. 1B and C, white arrows). For IT/R29, most HBEC-5i showed no adherent IE; however, occasional cells displayed a dense, clustered pattern of 10 to 20 adherent IE (Fig. 1D). During washing stages, rosette-like clusters of cells were seen binding to HBEC-5i; however, after fixation with glutaraldehyde, the uninfected erythrocytes were mostly lost, and only IE remained adherent. The disintegration of IT/R29 rosettes following treatment with glutaraldehyde has been noted previously (J. A. Rowe, unpublished data).

Adhesion of IT/R29 IE was also tested in ring-stage start assays with two primary endothelial cell lines, HPMEC and HBMEC. Binding was seen with both primary cell lines (Fig. 1E), although binding was significantly lower to HBMEC than to HBEC-5i (*P* = 0.009 by one-way ANOVA with Tukey's *post hoc* test). With each primary cell line, the bound IE occurred in clusters similar to those seen with HBEC-5i.

### Bound IT/R29 IE are recognized by antibodies against IT4var09 domains.

**FIG 2:**
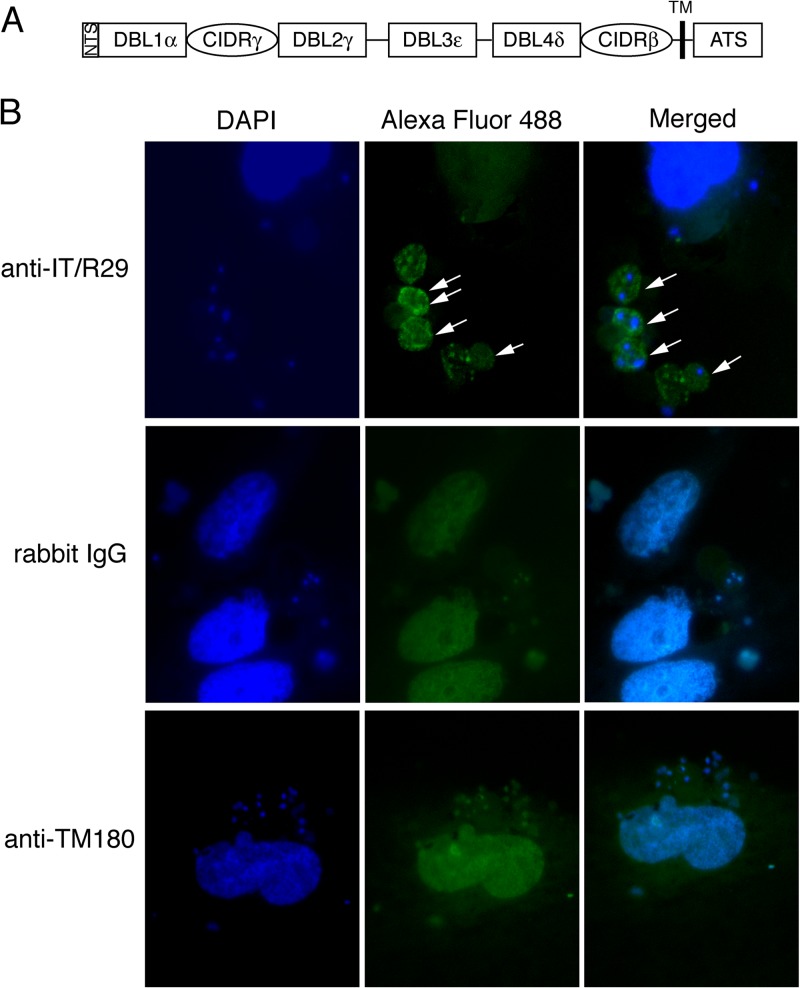
Antibodies against the IT4var09 PfEMP1 variant recognize IE bound to HBEC-5i. (A) Diagram of the IT4var09 PfEMP1 variant expressed by IT/R29 rosetting IE. The extracellular region is composed of multiple Duffy binding-like (DBL) and cysteine-rich interdomain regions (CIDR). TM, transmembrane region; ATS, acidic terminal segment. (B) Immunofluorescence assay (IFA) of IT/R29 IE adhering to HBEC-5i. Ring-stage IE at 5% parasitemia were incubated overnight with HBEC-5i in 8-well chamber slides. The following day the nonadherent IE were washed off and the slides fixed in acetone-methanol prior to immunostaining. Rabbit polyclonal antibodies against the IT4var09 NTS-DBL1α domain (anti-IT/R29) at 1:5,000 dilution were incubated for 1 h and then washed prior to detection with Alexa Fluor 488 goat anti-rabbit IgG at 1/1,000 dilution (green). The nuclei of the cells were stained with 1 μg/ml 4,6-diamidino-2-phenylindole (DAPI; blue; large nuclei are HBEC-5i, small nuclei are IE). Negative controls were IgG from a nonimmunized rabbit (rabbit IgG) and rabbit polyclonal antibodies to the NTS-DBL1α domain of an irrelevant PfEMP1 variant TM180var1 (anti-TM180) tested at 1/5,000 dilution. Slides were viewed with a 100× objective using a Leica DM LB2 fluorescence microscope, and images were taken at consistent exposure for all antibodies. Adherent IE staining with the IT4var09 antibodies are shown by white arrows.

Rosetting IT/R29 IE express the PfEMP1 variant IT4var09 (Fig. 2A) (18) (called R29var1 in earlier publications [15]) with the N-terminal domain (NTS-DBL1α) of this variant mediating binding to erythrocytes (15). To determine if the HBEC-5i-bound IE are indeed expressing IT4var09, the adherent cells were tested by immunofluorescence assay (IFA) on fixed cells with IT4var09-specific rabbit polyclonal IgG antibodies. The anti-IT4var09 NTS-DBL1α antibody showed bright positive staining of 100% of adherent IE (Fig. 2B, top row), while the negative-control wells with nonimmunized rabbit IgG (Fig. 2B, middle row) and antibodies to an irrelevant PfEMP1 (TM180var1 NTS-DBL1α [28]) (Fig. 2B, bottom row) showed only faint diffuse background staining of both HBEC-5i cells and IE. Antibodies to the IT4var09 DBL2γ domain were also tested and gave positive staining similar to that of the IT4var09 NTS-DBL1α antibodies (not shown). The IT4var09 PfEMP1 antibodies gave a smoother fluorescent pattern using fixed cells, as shown here, than the punctate pattern seen previously using live cells (18, 28). It may be that during the live cell IFA, PfEMP1 molecules become clustered due to antibody cross-linking. Alternatively, in fixed cells the antibodies may access internal as well as surface PfEMP1, giving a different staining pattern. The data shown in Fig. 2 indicate that the HBEC-5i-binding IE are expressing the IT4var09 variant, shown previously to mediate rosetting (15); therefore, IT4var09-expressing IE are capable of both rosetting and endothelial cell adhesion.

Although not examined in detail, two other P. falciparum rosetting strains, TM284R^+^ (expressing the TM284var1 variant [32]) and IT/PAR^+^ (expressing the IT4var60 variant [28]) were examined in standard and ring-stage start adhesion assays with HBEC-5i. For TM284R^+^, binding was seen in ring-stage start assays only, whereas for the knobless IT/PAR^+^ strain, no binding was seen in either assay.

### Antibodies against IT4var09 can inhibit and reverse adhesion to HBEC-5i.

**FIG 3:**
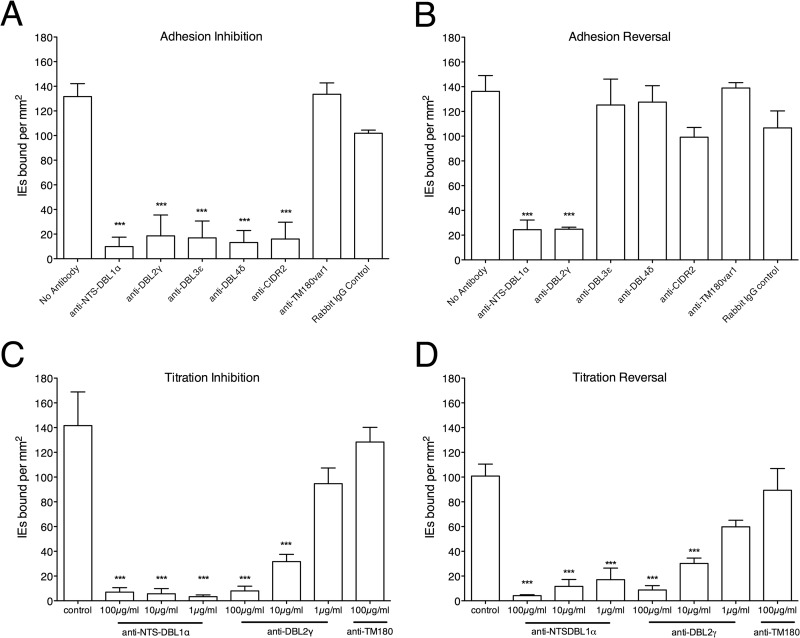
IT4var09 antibodies inhibit and reverse IT/R29 IE adhesion to HBEC-5i. (A) Antibodies against IT4var09 domains at 100 μg/ml inhibited adhesion of IT/R29 IE to HBEC-5i, whereas negative-control antibodies (nonimmunized rabbit IgG and TM180var1 antibodies) did not. (B) Antibodies against IT4var09 domains were tested at 100 μg/ml to determine their ability to reverse adhesion of IT/R29 IE to HBEC-5i. Only NTS-DBL1α and DBL2γ antibodies were capable of significantly reversing adhesion. NTS-DBL1α and DBL2γ antibodies showed a dose-dependent effect in both inhibition (C) and reversal (D) experiments. Data shown are the means and standard errors from three independent experiments in all cases. Statistical significance was determined by one-way ANOVA with Tukey's *post hoc* test. ***, *P* < 0.005.

To determine whether PfEMP1 is responsible for IT/R29 IE adhesion to HBEC-5i cells, rabbit polyclonal antibodies against specific domains of the IT4var09 PfEMP1 variant (Fig. 2A) were tested for their ability to both inhibit and reverse adhesion. For adhesion inhibition (Fig. 3A), antibodies against specific domains at 100 μg/ml of total IgG were added prior to the assay and coincubated with both ring-stage IE and HBEC-5i cells overnight. Antibodies against all domains were found to inhibit adhesion to HBEC-5i, while antibodies to an irrelevant PfEMP1 variant from another strain (anti-TM180var1 [28]), added as a negative control, failed to significantly alter the adhesion of IT/R29 IE (Fig. 3A).

Antibodies were then tested for their ability to reverse existing adhesion by adding them after 24 h of IT/R29 and HBEC-5i coculture (Fig. 3B). Both anti-NTS-DBL1α and anti-DBL2γ significantly reversed adhesion compared to the untreated control (*P* < 0.005 by one-way ANOVA with Tukey's *post hoc* test), while antibodies against the remaining domains DBL3ε, DBL4δ, and CIDR2β failed to significantly alter adhesion (Fig. 3B). The effects of the anti-NTS-DBL1α and anti-DBL2γ antibodies were dose dependent, and antibodies against the NTS-DBL1α domain inhibited and reversed adhesion at lower concentrations than the anti-DBL2γ antibodies (Fig. 3C and D). The IT4var09 antibodies did not agglutinate the IE under the conditions used in the assays described above.

### Adhesion of IT/R29 IE to HBEC-5i cells is heparan sulfate dependent.

**FIG 4:**
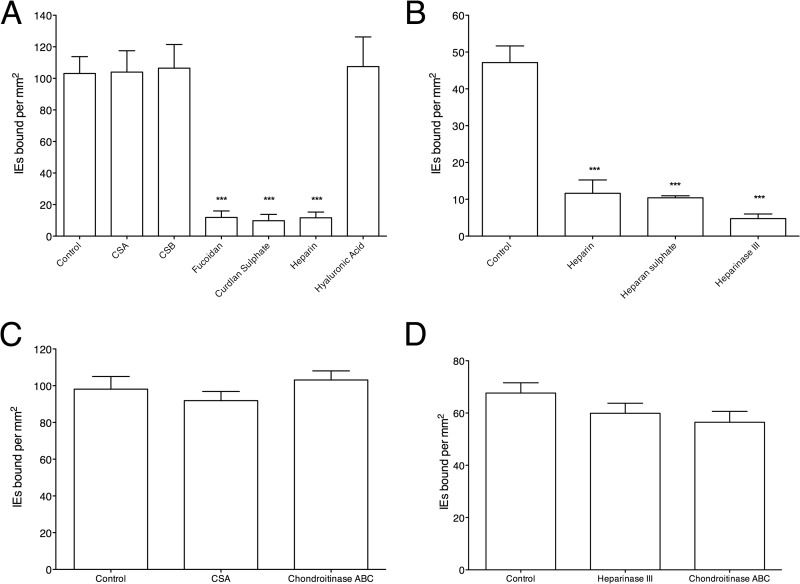
Adhesion of IT/R29 IE to HBEC-5i is heparan sulfate sensitive. (A) The ability of sulfated glycoconjugate compounds to reverse IT/R29 IE adhesion to HBEC-5i was tested by incubating cocultured IT/R29 IE and HBEC-5i for 1 h with 100 μg/ml of compound. Slides were then washed by gravity, fixed with 2% glutaraldehyde, stained with Giemsa, and assessed by microscopy (10 fields, 40× objective). The sulfated glycoconjugate compounds were dissolved in PBS, and the negative control was the addition of an equivalent volume of PBS alone with no added compound. (B) The ability of 100 μg/ml of heparin and heparan sulfate to reverse IT/R29 IE adhesion was performed as described for panel A. The effect of heparinase III on adhesion reversal was studied by incubation of cells with 0.2 IU/ml of enzyme for 2 h to remove heparan sulfate residues. Cells were washed, stained, and assessed by microscopy as described for panel A. Data shown are the means and standard errors from at least three independent experiments for panels A and B. (C) Soluble CSA (100 μg/ml for 2 h) and chondroitinase ABC enzyme (0.5 IU/ml for 2 h) were tested for their ability to reverse IT/R29 adhesion as described for panels A and B. (D) Heparinase III (0.2 IU/ml for 2 h) and chondroitinase ABC (0.5 IU/ml for 2 h) were tested for their effect on HB3-HBC adhesion. For panels C and D, the negative control was addition of an equivalent volume of PBS alone with no added compound/enzyme, and in each case means and standard errors from 2 independent experiments are shown. Statistical significance was determined by one-way ANOVA with Tukey's *post hoc* test. ***, *P* < 0.005.

Previous work had suggested that the glycosaminoglycan heparan sulfate on endothelial cells can act as a receptor for adhesion of parasite strain FCR3S1.2 (13). Furthermore, previous experiments on IT/R29 rosetting parasites had demonstrated a sensitivity to sulfated glycoconjugates, whereby rosettes were successfully disrupted by soluble heparin, fucoidan, and curdlan sulfate but were not affected by CSA, CSB, or hyaluronic acid (20, 21). Therefore, the role of sulfated glycoconjugates in IT/R29 binding to HBEC-5i was investigated further. A panel of sulfated glycoconjugates, including heparin, fucoidan, curdlan sulfate, CSA, CSB, and hyaluronic acid, were tested for their ability to reverse the adhesion of IT/R29 IE to HBEC-5i. Mirroring the effect of sulfated glycoconjugates on rosetting, we found that heparin, fucoidan, and curdlan sulfate all reversed IT/R29 IE binding to HBEC-5i, whereas CSA, CSB, and hyaluronic acid had no effect on binding (Fig. 4A).

Having shown that heparin inhibits IT/R29 HBEC-5i binding, the role of the physiologically relevant endothelial cell heparan sulfate proteoglycan was investigated (heparin is a highly sulfated form of heparan sulfate found only in mast cells). The addition of soluble heparan sulfate significantly reversed binding of IE, similar to the effect of heparin (Fig. 4B). To further investigate the role heparan sulfates play in mediating adhesion, HBEC-5i cells were enzymatically treated to selectively cleave the heparan sulfate molecules from the surface of the cells. This treatment significantly reversed the adhesion of IT/R29 IE by 84% (Fig. 4B), indicating that heparan sulfate molecules are required for adhesion of IT/R29 IE to HBEC-5i. Adhesion of IT/R29 to HBEC-5i cells was not significantly reduced after control treatment with the chondroitinase ABC enzyme, which removes chondroitin sulfates (Fig. 4C). To rule out the heparinase III enzyme eliciting an effect upon the parasite and not the HBEC-5i cells, a control parasite line, HB3-HBEC, selected for adhesion to HBEC-5i cells (24, 25), was pretreated with heparinase III or chondroitinase ABC before use in a standard pigmented trophozoite-stage adhesion assay. Neither enzyme was capable of significantly reducing adhesion of HB3-HBEC to HBEC-5i cells (Fig. 4D).

### The PfEMP1 variant IT4var09 possesses multiple heparin-binding domains.

**FIG 5:**
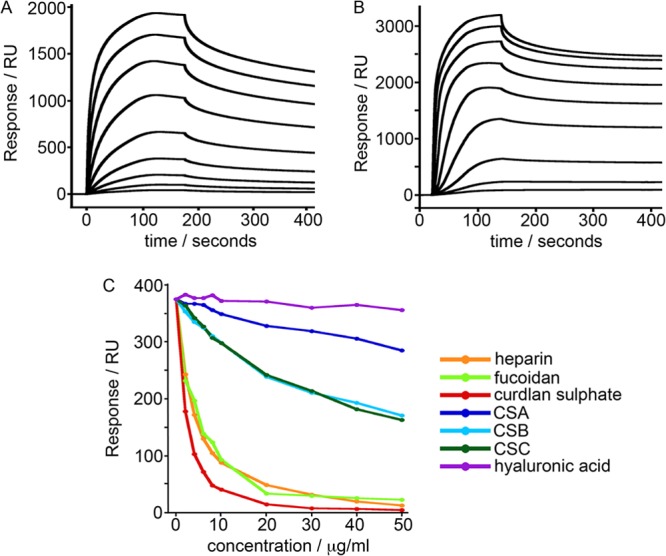
IT4var09 NTS-DBL1α and DBL2γ domains bind heparin, and the binding is inhibited by sulfated glycoconjugates. (A) SPR signal for the binding of IT4var09 NTS-DBL1α from a maximum concentration of 4 μM to a heparin-coated surface. (B) SPR signal for the binding of DBL2γ from a maximum concentration of 4 μM to a heparin-coated surface. (C) Competition experiments in which the IT4var09 DBL2γ domain was incubated with different concentrations of glycoconjugates before assessment of binding to a heparin-coated surface.

The data described above suggest that IT/R29 IE bind to HBEC-5i through an interaction with heparan sulfates and show that antibodies to the NTS-DBL1α and DBL2γ domains of the IT4var09 PfEMP1 reverse adhesion. The antibody reversal data suggest that the NTS-DBL1α and DBL2γ domains of IT4var09 are directly involved in binding to heparan sulfate. To test this hypothesis, heparin binding by each of the four DBL domains from the IT4var09 PfEMP1 variant (Fig. 2A) was tested by SPR. While the DBL3ε and DBL4δ domains showed little or no binding to a heparin-coated surface, both NTS-DBLα and DBL2γ bound strongly (Fig. 5A and B). Equilibrium analysis of saturated responses due to binding of different concentrations of NTS-DBL1α and DBL2γ showed half-maximal binding at a concentration of ∼0.5 μM in both cases. Attempts were also made to fit kinetic data to a variety of binding models, but the dissociation kinetics were complex and did not fit to a simple 1:1 model, as previously observed for chondroitin sulfate proteoglycan- binding domains from other PfEMP1 proteins, suggesting the possibility of multiple modes of binding (33).

To further compare the heparin binding of the IT4var09 DBL2γ domain shown by SPR to the adhesion capabilities of IT/R29 IE, the ability of various sulfated glycoconjugates to block heparin binding by DBL2γ IT4var09 was assessed (Fig. 5C). The soluble compounds that block adhesion of IT4var09 DBL2γ to heparin by SPR (heparin, fucoidan, and curdlan sulfate) mirror those that also disrupt rosettes (20, 21) and reverse adhesion of IT/R29 IE to HBEC-5i cells (Fig. 4A), whereas the nonblocking or poorly blocking compounds (CSA, CSB, CSC, and hyaluronic acid) have no reversal effect on rosetting (20) or HBEC-5i binding of IT/R29 IE (Fig. 4A). These data suggest that the heparin binding of the IT4var09 DBL2γ domain demonstrated by SPR is physiologically relevant to the binding capabilities of IT/R29 IE.

### CR1 does not play a role in endothelial cytoadherence of IT/R29 IE.

**FIG 6:**
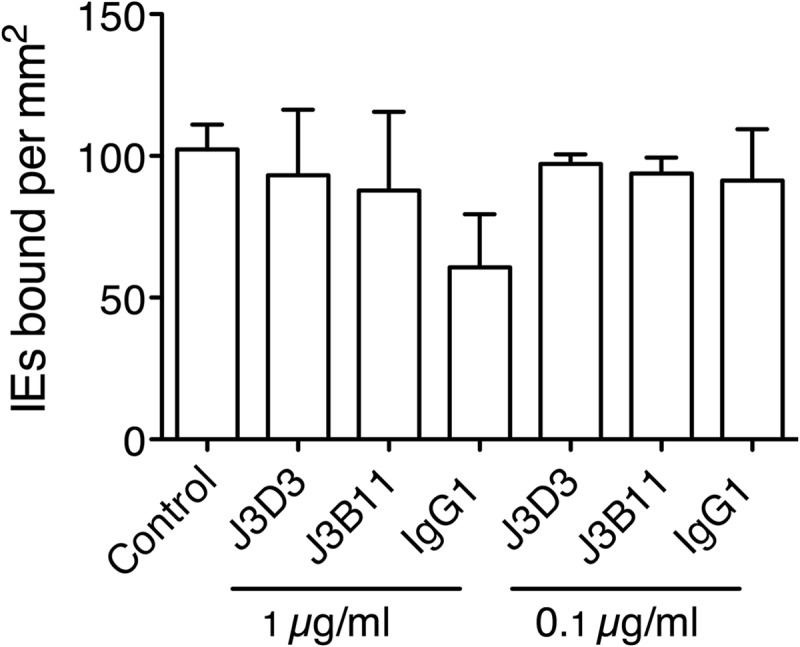
Adhesion of IT/R29 IE to HBEC-5i is not CR1 dependent. Mouse monoclonal antibodies to CR1 were tested for their ability to inhibit adhesion of IT/R29 IE to HBEC-5i, and no significant inhibition was seen. Data shown are the means and standard errors from two independent experiments.

Rosetting of IT/R29 parasites is dependent on CR1 on uninfected erythrocytes (15, 19). To explore whether CR1 also could play a role in endothelial cytoadherence, we tested the ability of a rosette-disrupting CR1 MAb, J3Bll, to inhibit adhesion of IT/R29 IE to HBEC-5i. J3B11, the control MAb J3D3 (a non-rosette-disrupting CR1 MAb), and an isotype control failed to significantly inhibit adhesion of IT/R29 IE to HBEC-5i (Fig. 6), indicating that CR1 does not play a role in IT/R29 cytoadherence. These results are consistent with previous data showing that CR1 could not be detected on HBEC-5i or HPMEC (34).

## DISCUSSION

Despite the importance of rosetting as a parasite adhesion phenotype linked to life-threatening malaria, the ability of rosetting IE to contribute to P. falciparum sequestration by binding directly to endothelial cells remains poorly understood. The data presented here show that IE of the rosetting IT/R29 strain can bind to microvascular endothelial cells *in vitro*. Furthermore, we identified both the host receptor (heparan sulfate) and the parasite ligand (PfEMP1) and showed that two domains of the IT04var09 PfEMP1 variant (NTS-DBL1α and DBL2γ) are involved in binding interactions. Adhesion of IT/R29 IE was only apparent when the parasites were cocultured with HBEC-5i from ring stage (before rosetting begins), probably because at later stages, the presence of rosettes interferes with interaction of IE with the endothelial cell surface. The pattern of adhesion observed was different from that seen with other nonrosetting adherent parasite strains, such as FCR3-CSA (which binds CSA) and HB3-HBEC (which binds an unknown endothelial cell receptor). For the latter two strains, IE bind in an even distribution over a lawn of endothelial cells (Fig. 1B and C), while for IT/R29 binding was clustered, with a small number of endothelial cells showing dense binding of multiple IE (Fig. 1D). It is unclear whether the clustered distribution of IT/R29 IE is due to variation in heparan sulfate expression levels or sulfation patterns on the HBEC-5i cells or is a “seeding” effect, whereby one adherent IE attracts others, partly due to their interactions with the HBEC-5i cells and partly due to the rosetting IE binding other IE.

Previous work has identified the NTS-DBL1α domain of the IT4var09 PfEMP1 variant as mediating rosetting via interactions with CR1 on uninfected red cells (15, 19). Interestingly, our results indicate that this PfEMP1 domain is also capable of mediating adhesion to endothelial cells, as antibodies directed against it could both inhibit and reverse adhesion in a dose-dependent manner (Fig. 3), and direct binding of NTS-DBL1α to heparin was demonstrated by SPR (Fig. 5). These data strongly suggest this domain has a dual adhesive phenotype, in that it mediates both rosetting and cytoadherence. However, rosetting of IT/R29 IE involves CR1 as a receptor, whereas our data indicate that endothelial cytoadherence occurs via heparan sulfate (Fig. 4) and does not involve CR1 (Fig. 6). IT/R29 rosetting is not thought to involve heparan sulfate-like molecules on erythrocytes, because treatment of erythrocytes with heparinase enzyme does not reduce IT/R29 rosetting (35). Therefore, our data suggest that distinct PfEMP1 domains within a single variant can interact with different receptors to bring about adhesion to alternative human cell types. It remains possible that interactions with heparan sulfate-like molecules on erythrocytes contribute to strengthening IT/R29 rosettes, even if they are not essential for rosette formation.

Our investigations also identified a second PfEMP1 domain involved in cytoadherence, the DBL2γ domain. In contrast to NTS-DBL1α, antibodies raised against DBL2γ of IT4var09 did not inhibit or reverse rosetting (18) but did successfully inhibit and reverse adhesion to HBEC-5i cells (Fig. 3). These data suggest that the DBL2γ domain is involved in mediating endothelial adhesion but does not play a major role in rosetting. It is possible that the DBL2γ antibody can reverse cytoadhesion but not rosetting, because the binding of IE to endothelial cells is weaker than the binding to uninfected erythrocytes in rosettes.

It has been suggested previously that demonstration of binding of individual PfEMP1 domains to sulfated glycoconjugate molecules *in vitro* was not necessarily predictive of meaningful interactions between native full-length PfEMP1 on the surface of IE and sulfated glycoconjugates (36). In our study, the results from single-domain SPR experiments showed that IT4var09 NTS-DBL1α and DBL2γ domains bind heparin, and that the interaction is inhibited by soluble heparin, fucoidan, and curdlan sulfate but not by CSA, CSB, CSC, or hyaluronic acid. These results exactly mirror the effect of sulfated glycoconjugates on IT/R29 rosette disruption (20) and IT/R29 binding to HBEC-5i (Fig. 4A), giving confidence that in this case, the results of SPR with individual domains are biologically meaningful. Given the distinct receptors involved in IE cytoadherence to HBEC-5i and rosetting, it seems likely that the cytoadherence-blocking effect of heparin and other sulfated glycoconjugates (Fig. 4) is due to direct blockade of the receptor-ligand interaction, whereas the rosette-inhibiting effects of these compounds (20, 21) may occur due to steric or electrostatic effects, as suggested previously (37).

Our initial studies on rosetting IE interactions with endothelial cells using standard assays with pigmented trophozoite-stage parasites were unsuccessful (Fig. 1), most likely due to the presence of rosettes blocking interactions between IE surface molecules and endothelial cell receptors. The inhibitory effect of preexisting rosettes on adhesion is supported by previous work of Handunnetti and colleagues, who used the CD36-dependent rosetting laboratory parasite strain Malayan Camp and showed that IE could bind to purified CD36 protein or C32 melanoma cells but only adhered well if rosettes were disrupted prior to the assay (38). However, most P. falciparum rosetting isolates are not CD36 dependent (19, 39, 40), so the broader significance of the findings reported with the Malayan Camp strain are unclear. The possibility of adhesion of rosetting IE to endothelial cells was also suggested by Udomsangpetch et al., who demonstrated binding to HUVEC via an unknown receptor (41). However, HUVECs show many differences in receptor expression and biological functions compared to microvascular endothelial cells (42), and the significance of these findings in terms of microvascular adhesion was unclear. The more recent work by Vogt and colleagues (13) did use a microvascular human lung endothelial cell line as well as HUVEC, but as outlined in the introduction, it is likely that nonrosettting IE were studied. Therefore, our report represents the first clear demonstration, to our knowledge, that rosetting IE can bind directly to microvascular endothelial cells.

Further experiments will be necessary to test the physiological significance of the IE-endothelial cell interactions described here. The interactions between IT/R29 IE and endothelial cells were relatively weak, as gentle gravity washes were required to visualize the binding. We did attempt to study IT/R29 IE interactions with HBEC-5i under flow conditions and saw rolling of rosetting IE with a low level of adhesion (3 to 4 per field of 200 μm^2^; data not shown) at shear stresses similar to those found in the microvasculature *in vivo* (0.5 to 1.0 dyn/cm^2^) (43). However, as described above for the static assays, the flow experiments are complicated by the presence of rosettes potentially blocking interaction between IE surface molecules and endothelial cells, especially as large “multirosettes” form under flow conditions (44). Previous experiments using rosetting parasites in an *ex vivo* model suggested that rosettes are disrupted in the arterial side of the circulation (due to high shear forces) but then would reform in the venous side of the circulation, with rosetting and endothelial cytoadherence occurring simultaneously in postcapillary venules (45). The variation in vessel size and shear stresses experienced under pulsatile blood flow *in vivo* is difficult to mimic *in vitro*, and further *ex vivo* experiments may be required to explore more fully the potential of rosetting parasites to contribute to sequestration via direct cytoadherence to endothelial cells. It may be that rosetting IE-endothelial cell interactions are not a major primary cause of sequestration *in vivo*, but that such interactions could contribute to cytoadherence in areas of low or stagnant flow secondary to sequestration of nonrosetting IE. Another possibility is that rosetting IE make a major contribution to sequestration *in vivo* via adhesion to other nonrosetting IE that are bound to endothelial cells. Our attempt to use MACS-purified IE in adhesion assays was unsuccessful because of the massive aggregates of IE that rapidly occur when uninfected erythrocytes are removed from the culture due to strong binding of rosetting IE to other IE. Clinical isolates are usually multiclonal, and even within a single clone, multiple different *var* genes are expressed, giving a parasite population with diverse binding characteristics. It may be that *in vivo*, rosetting can contribute to sequestration both via direct binding of rosetting IE to microvascular endothelium in areas of stagnant flow and also more widely via binding of rosetting IE to cytoadherent nonrosetting IE.

Although the precise way in which rosetting contributes to sequestration is unknown, it remains clear that rosetting is a parasite phenotype linked to pathogenicity. Evidence includes the consistent association between parasite rosette frequency and disease severity in sub-Saharan Africa (10–12), the virulence of rosetting parasites in an animal model (46), and the proven ability of human erythrocyte polymorphisms that reduce the ability of P. falciparum to form rosettes to confer significant protection against life-threatening malaria (47, 48). The work done here emphasizes the potential for rosette-disrupting interventions that could be used as adjunctive therapies against severe malaria. Furthermore, the findings that the same set of sulfated glycoconjugate compounds successfully reverses both rosetting and adhesion to HBEC-5i cells (Fig. 4) and that antibodies to the NTS-DBL1α domain of IT4var09 reverse both rosetting and adhesion to HBEC-5i cells (Fig. 3) suggest that it will be possible to identify therapeutic agents that will disrupt both rosetting and endothelial adhesion. A heparin derivative lacking anticoagulant effects (49) is currently undergoing clinical trials, and the data presented here support the potential of such compounds as adjunctive therapies for severe malaria.

Taken together, the data shown here illustrate the importance of the N terminus of the PfEMP1 molecule in adhesion of IT/R29 IE to endothelial cells, as well as rosetting as shown previously, and support the identification of heparan sulfate as the host endothelial cell receptor. This demonstration of the dual adhesion of rosette-capable IE to endothelial cells further enhances the argument for the role of rosetting in severe malaria and pathogenicity.
